# Basic assessment on adding platelet measurement to legal health checkup in Japan: A cross-sectional and 20-year longitudinal study

**DOI:** 10.3389/fpubh.2023.1106831

**Published:** 2023-04-03

**Authors:** Kayoko Kasuya, Kota Fukai, Yuuya Watanabe, Yuko Furuya, Shoko Nakazawa, Toru Honda, Takeshi Hayashi, Toru Nakagawa, Masayuki Tatemichi, Masaaki Korenaga

**Affiliations:** ^1^Hitachi Health Care Center, Hitachi, Japan; ^2^Department of Preventive Medicine, School of Medicine, Tokai University, Isehara, Japan; ^3^Occupational Hygiene and Promotion Center, Hitachi Ltd., Tokyo, Japan; ^4^Hepatitis Information Center, The Research Center for Hepatitis and Immunology, National Center for Global Health and Medicine, Ichikawa, Chiba, Japan

**Keywords:** platelet count, legal health checkups, FIB-4 index, viral hepatitis, fatty liver

## Abstract

**Background:**

In Japan, health checkups for workers are legally compulsory. Considering legal health checkup items are important for Japanese workers' health problems. To date, the legal health checkup items for blood cell counts include only red blood cell counts and hemoglobin but not platelet counts. This study aimed to investigate the significance of measuring platelets in workers by showing the association between the FIB-4 index (FIB-4), which can be easily calculated from factors including platelet counts and viral hepatitis infection.

**Method:**

Both cross-sectional and longitudinal analyses were conducted on the comprehensive medical examinations of male workers. In fiscal year (FY) 2019, a logistic regression model was applied to 12,918 examinees. For 13,459 examinees (mean age = 47.5 ± 9.3 SD), FY2000 was set to be followed until FY2019. A total of 149,956 records between FY2000 and FY2019 were analyzed cross-sectionally, and 8,038 men who were consecutively examined to FY2019 at the longest were analyzed longitudinally. Receiver operating characteristic (ROC) curve–area under the ROC curve (ROC–AUC) and Cox proportional methods were used to examine the association between platelet-related indices and viral hepatitis infection.

**Results:**

Logistic regression showed that the risk of FIB-4 ≥ 2.67 was mostly associated with hepatitis C virus antibody (HCVAb) positivity [odds ratio (OR) = 2.51, 95% confidence interval (CI) = 1.08–5.86], while negatively associated with body mass index (BMI) (OR = 0.54, 95% CI = 0.30–0.97), and not associated with the presence of fatty liver. To detect HVC Ab positivity, ROC–AUC showed more effectiveness in FIB-4 than in the AST/ALT ratio (0.776, 95% CI = 0.747–0.773 vs. 0.552; 95% CI = 0.543–0.561). The Cox analysis showed that the risk of FIB-4 ≥ 2.67 was closely associated with hepatitis B virus surface antigen (HBsAg) [hazard ratio (HR) = 3.1, 95% CI = 2.0–4.6] and HCV Ab positivity (HR = 3.2, 95% CI = 2.0–5.0).

**Conclusion:**

Our results suggest that it might be worth considering that usage of information on platelets in legal health checkups could be some help not to overlook workers with hepatitis virus carriers as a complementary countermeasure, although further investigations are needed into its practical application.

## Introduction

In Japan, health checkups for workers are legally compulsory.^1^ Considering legal health checkup items are important for Japanese workers' health problems. Thus, the items are reviewed every 10 years as a policy.

To date, the legal health checkup items to determine blood cell counts include only red blood cell counts and hemoglobin, but not platelet counts (see text footnote^1^), as well as legal special health checkups against metabolic syndrome.^2^ Platelets, generally, fluctuate in association with hematological disorders, such as hyperplasia and hypoplasia, but their roles in health examinations have not been previously examined.

Japanese labor law requires employers to consider the working conditions of their employees based on their health status, and the law specifies the items to be examined by the employers. The only legal items related to blood cell counts are those related to anemia. Practically, however, the majority of blood cell counts, including red blood cell and hemoglobin counts, are measured by devices that calculate platelets automatically and simultaneously. Despite this, these platelet results are intentionally deleted and unused because there is no legal requirement in Japan for these data. This is because platelet counts are known to decrease due to liver–spleen dysfunction, especially liver fibrosis (1). The progression of liver fibrosis is the best predictor of liver disease progression. In recent years, several non-invasive scoring systems have been developed based on clinical and biochemical variables to assess liver fibrosis (2, 3). These scoring systems often include platelet counts (2, 3). The legal items in Japan should be reviewed as the development of medicine, and the platelet information described earlier should be utilized.

To investigate the significance of information on platelet counts for general workers' health, we considered the FIB-4 index (FIB-4) as one candidate, which was developed as a simple index for liver fibrosis. FIB-4 can easily be calculated based on platelets in addition to aspartate transaminase (AST) and alanine transaminase (ALT), which are already legal health checkup items (4). Although FIB-4 has been intensively studied in clinical settings, only two studies were reported in general settings and its significance is largely unknown (5, 6).

Of the total 30 million legal health checkup recipients,^3^ ~4 million (13.3%) who received comprehensive health examinations such as “Ningen dock” have platelet data in Japan.^4^ The research question is whether indices such as FIB-4 are valuable health examination metrics using a cross-sectional and 20-year longitudinal dataset from general workers' health examinations. The objective of this study was to examine the association between FIB-4 and viral hepatitis infection. By investigating these associations, the data will provide a basis for reconsidering the policy to consider legal health checkup items.

## Methods

### Study design

This study was conducted at a health center belonging to a member of a group of large companies. Employees and their spouses from ~30 affiliated companies (30,000 employees) freely selected the timing and health center where they underwent comprehensive health examinations. Details were described previously (7–11). In this study, two datasets of comprehensive medical examinations were analyzed as two cross-sectional and one longitudinal study for the following years. First, the most current data of examinees at FY2019, 15,792 records, including 13,700 men and 2,092 women [mean age ± standard deviation (SD) = 53.0 ± 10.0], were obtained. The second, 16,408 examinees (men = 13,701 and women = 2,707; mean age = 47.8 ± 9.2) at FY2000 were obtained as a baseline and were followed up to FY2019 for every year, and then a total number of 183,477 records, including 159,081 men and 21,396 women were obtained.

**Figure 1:**
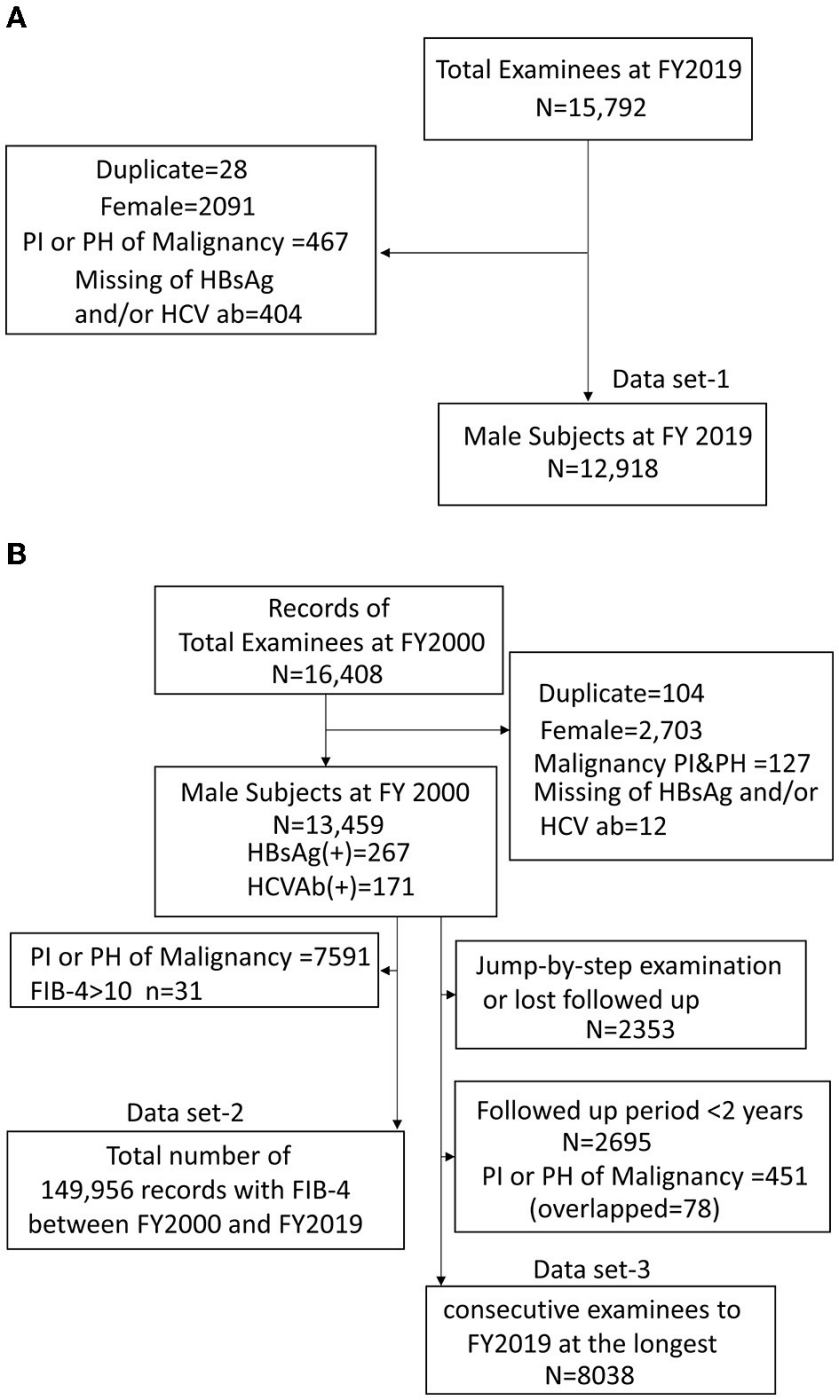
Flow diagram of the inclusion and exclusion of subjects on dataset-1 **(A)**, dataset-2,3 **(B)**. PI, present illness; PH, present history; HBsAg, hepatitis B virus surface antigen; HCVAb, hepatitis C virus antibody.

For this study, only men were enrolled because the number of women was small. The FIB-4 index of <1.3, 1.3–2.67, or ≥2.67 was considered as a low, moderate, or high risk for fibrosis, respectively (12, 13). To identify factors associated with FIB-4 ≥ 2.67, among 13,700 male examinees in FY2019, 12,918 subjects without a present or past history of malignancy were analyzed as dataset-1 (mean age ± SD = 53.1 ± 10.3). Subjects with a present or past history of malignancy were excluded because some use of anticancer drugs may be able to affect platelet count. A total number of 149,956 records that had available FIB-4 of subjects without a present or past history of malignancy between FY2000 and FY2019 for 20 years were analyzed as dataset-2 (mean age ± SD = 53.2 ± 9.1). Finally, to perform a longitudinal analysis of 13,459 male examinees at FY2000, 8,038 workers who were consecutively examined to FY2019 at the longest were analyzed as dataset-3 (mean age ± SD = 46.7 ± 8.4) having a mean follow-up period of 12.1 ± 6.0 years. The flow of these datasets is shown in Figure 1.

Information on present and/or past history of malignancy and alcohol consumption behavior was obtained from health questionnaires. Alcohol consumption was determined based on the information of daily alcohol intake (ethanol g/day) or daily alcohol intake × weekly drinking frequency as a continuous variable. The fatty liver disease information was obtained from ultrasonographic findings in FY2019.

### Definitions

Viral hepatitis was diagnosed (defined as positive) when the hepatitis B virus surface antigen (HBsAg) and/or HCVAb tests were positive. In addition, ALT and/or AST values ≥40 IU/L were defined as liver dysfunction.

### Fibrosis markers

We selected non-invasive fibrosis markers that consist of general health checkup items or platelet counts; FIB-4 index, AST to platelet ratio index (APRI), non-alcoholic fatty liver disease NAFLD score (NFS), and AST to ALT ratio (AAR) not including platelet counts (12, 14). The equations used are as follows (12):

(1) FIB-4 Index = Age (years) × AST (IU/L)/[platelet count (10^9^/l) × √ALT (IU/L)]
(2) APRI = [(AST/upper limit of normal)/platelet count (10^9^/l)] × 100
(3) NFS = −1.675 + 0.037 × Age (years) + 0.094 × BMI + 1.13 × IFG/diabetes (yes = 1, no = 0) + 0.99 × AST/ALT – 0.013 × platelet count (10^9^/l) −0.66 × albumin (g/dl)
(4) AAR = AST/ALT

### Statistical analysis

#### Identification of factors of FIB-4 ≥ 2.67

The factors of FIB-4 ≥ 2.67 were identified using a logistic model with the FY2019 dataset-1. Variables were selected from previous studies to show associations with NAFLD [obesity (BMI), abdominal circumference, present illness of diabetes, present illness of hypertension, and fatty liver] (8, 9), smoking (10), viral hepatitis (HBsAg and HCVAb), and alcohol consumption.

#### FIB-4 distribution and detection ability of viral hepatitis

Since age is included in the formula for FIB-4, the change was evaluated using the average and SD for each age, and the distribution was calculated by groups that were classified according to these FIB-4 reference values from dataset-1: <1.3 (low risk for fibrosis), 1.3–2.67 (mild risk for fibrosis), and ≥2.67 (high risk for fibrosis), based on results of fatty liver fibrosis (3, 4).

The viral hepatitis predictive ability of FIB-4, APRI, NFS, and AAR was examined using the receiver operating characteristic (ROC) curve–area under the ROC curve (ROC–AUC) and 95% confidence intervals (CIs) in dataset-2.

The Cox proportional hazard model was used to calculate the hazard ratio (HR) and 95%CI of HBsAg and HCVAb positivity for all outcomes of FIB-4 equal or exceeding 2.67 from FY2000 to FY2019 using dataset-3. In Model 1, HRs for HBsAg and HCVAb positivity were calculated using age, BMI, and alcohol consumption as co-variants in Model 1. In Model 2, since findings of “Fatty liver” are known to be the most important factors being associated with FIB-4 (3, 4), “Fatty liver” findings by ultrasonography in FY2019 were entered into the model. Thus, 2,510 male workers who could be followed up until FY2019 were subjected. All statistical analyses were conducted using IBM-SPSS ver. 26 (IBM, Armonk, NY, USA) and SAS ver. 9.4 (SAS Institute, Cary, NC, USA).

## Results

### Factors of FIB-4 ≥ 2.67

Table 1 shows factors associated with FIB-4 ≥ 2.67 by logistic analysis. Alcohol drinking and HCVAb positivity were significantly and positively associated, while BMI was negatively associated. The presence of fatty liver was not associated with FIB-4 ≥ 2.67. HCVAb positivity was mostly associated with FIB-4 ≥ 2.67 (OR = 2.52, 95% CI = 1.08–5.88).

**Table 1:** Factors associated with the FIB-4 index ≥ 2.67 by logistic analysis.

		**Multivariate**			
		**Odds ratio**	**95% CI**		***P***
Age (y)		1.17	1.15	1.19	< 0.001
Hypertension	No	1	Reference		
	Yes	1.09	0.83	1.44	0.53
Diabetes mellitus	No	1	Reference		
	Yes	0.94	0.65	1.37	0.74
Alcohol drinking	No drinker	1	Reference		
	<20 g/day	0.69	0.48	0.98	0.04
	20–40 g/day	1.85	1.34	2.56	< 0.001
	>40 g/day	2.19	1.32	3.63	0.00
Tobacco smoking	Never	1	Reference		
	Former	0.94	0.69	1.27	0.69
	Current	1.09	0.76	1.57	0.65
BMI (Kg/m^2^)	<20	1	Reference		
	20–22	0.69	0.42	1.15	0.16
	22–25	0.52	0.32	0.85	0.01
	≥25	0.54	0.30	0.97	0.04
Abdominal circumference (cm)	<85	1	Reference		
	≥85	0.98	0.68	1.42	0.93
HBsAg	–	1	Reference		
	+	2.08	0.85	5.11	0.11
HCVAb	–	1	Reference		
	+	2.51	1.08	5.86	0.03
Fatty liver^*^	No	1	Reference		
	Yes	1.36	0.85	2.15	0.20

* Detected by abdominal ultrasonography.

CI, confidence interval; BMI, body mass index; HBsAg, hepatitis B virus surface antigen; HCVAb, hepatitis C virus antibody.

Table 2 shows the descriptive data between hepatitis positivity and the status of ALT and/or AST for each age group in a cross-section of dataset-1. Among current examinees in FY2019, 86.8% (158/182) of subjects with viral hepatitis showed within the normal range of AST and ALT, and the hepatitis positivity rate was high in association with normal liver function. In addition, the ability to detect hepatitis by AST and ALT was evaluated using the ROC curve and ROC–AUC. The ROC–AUC of AST was 0.503 (95% CI = 0.464–0.543), and that of ALT was 0.452 (95% CI = 0.409–0.494).

**Table 2:** HBsAg or HCVAb test positive cases and status of liver function (ALT or AST) at FY 2019.

		***n* = 12,918**
Age	(Mean ± SD)	53.1 ± 10.3
BMI	(Mean ± SD)	24.4 ± 3.5
Liver function test		HBsAg/HCVAb positivity
AST < 40 and ALT < 40	Rate to total	1.5% (158/10,534)
	Rate to positive case	86.8% (158/182)
AST **≥** 40 and ALT < 40	Rate to total	0.9% (1/109)
	Rate to positive case	0.5% (1/182)
AST < 40 and ALT **≥** 40	Rate to total	1.0% (14/1,360)
	Rate to positive case	7.7% (14/182)
AST **≥** 40 and ALT **≥** 40	Rate to total	1.3% (9/733)
	Rate to positive case	4.9% (9/182)
Total (no. of positive/total)	Rate to total	1.4% (182/12,918)

HBsAg, hepatitis B virus surface antigen; HCVAb, hepatitis C virus antibody; BMI, body mass index; AST, aspartate aminotransferase; ALT, alanine aminotransferase.

### Fibrosis markers and viral hepatitis

**Figure 2:**
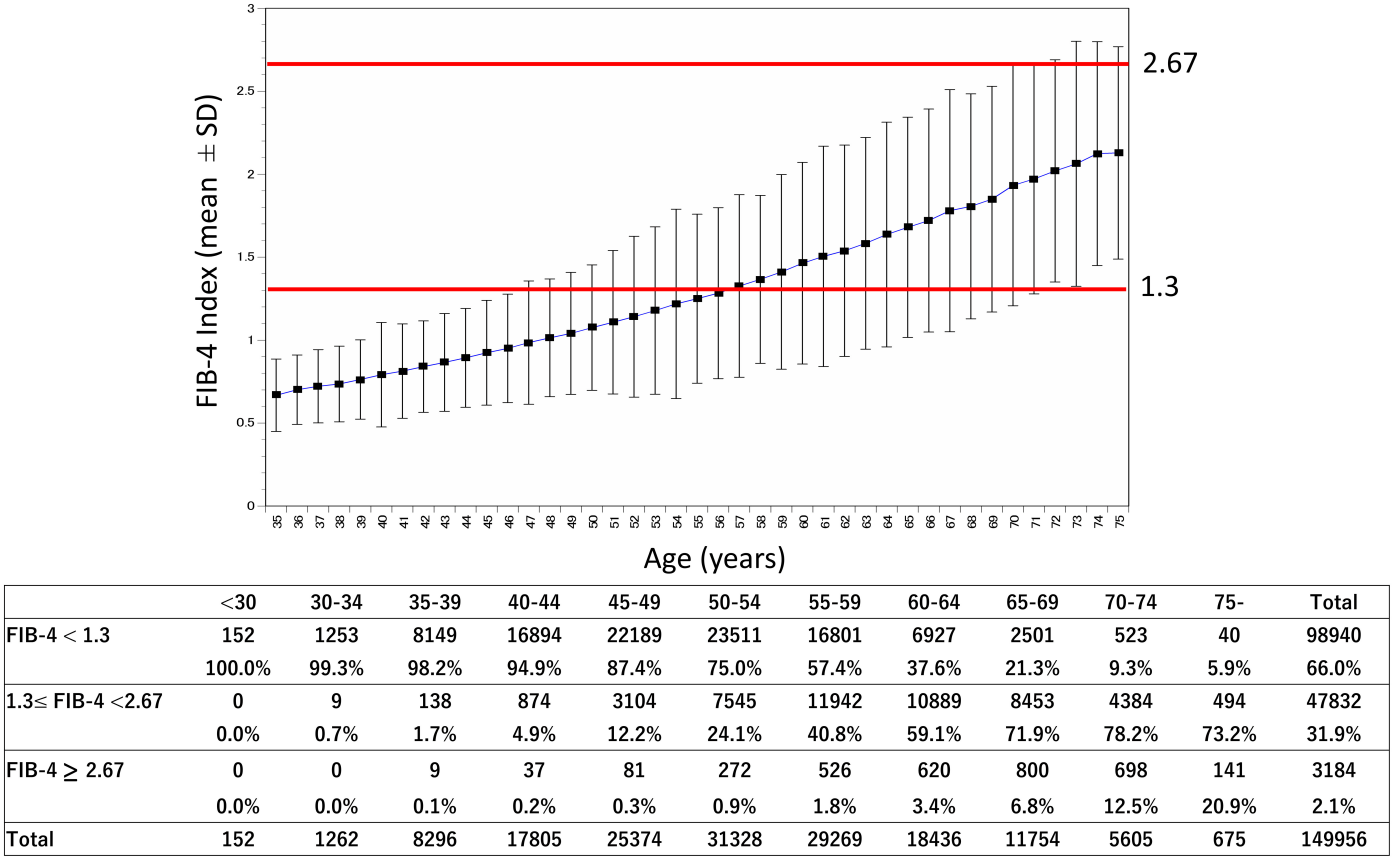
Age-related change of the FIB-4 index and rate of categories. The mean and SD of the FIB-4 index were shown by age from a population of 149,959 from FY2019 to FY2000. In addition, the number and rate by category of the FIB-4 index [<1.30 (low risk for fibrosis), 1.30–2.67 (mild risk for fibrosis), ≥2.67 (high risk for fibrosis)] are shown. FY, fiscal year; SD, standard deviation.

Figure 2 shows changes in FIB-4 by age and distributed in ranges: <1.30, 1.30–2.67, and ≥2.67. For those under 65 years of age, high FIB-4 (≥2.67) was observed in <2.2% of the population.

**Figure 3:**
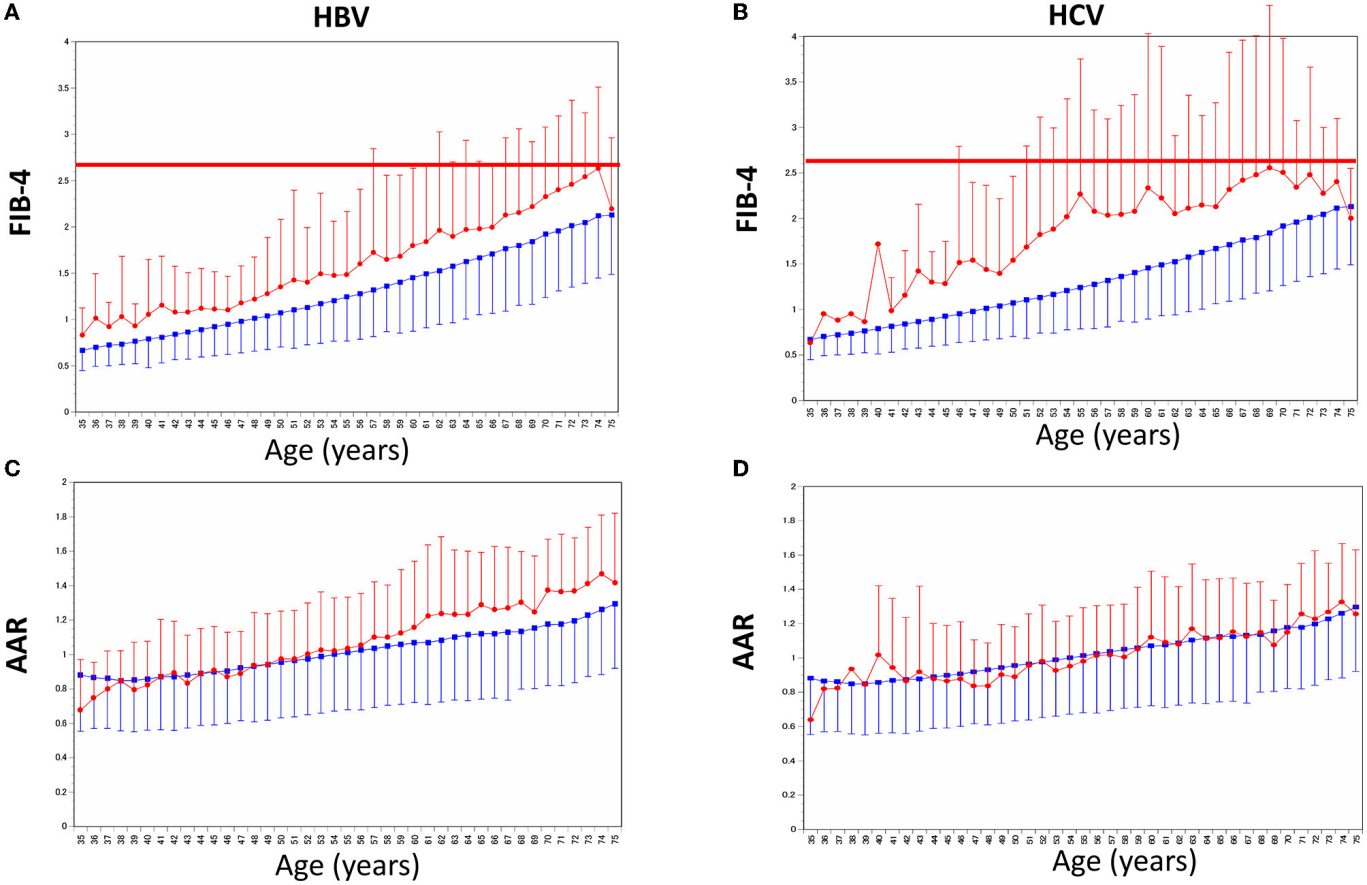
Age-related changes of the FIB-4 index and AAR by age between HBV/HCV positive and negative. **(A)** Mean and SD of the FIB-4 index were shown by age and HBV Ag positivity. **(B)** Mean and SD of the FIB-4 index were shown by age and HCVAb positivity. **(C)** Mean and SD of AAR were shown by age and HBV Ag positivity. **(D)** Mean and SD of AAR were shown by age and HCVAb positivity. Red and blue indicate positive and negative, respectively. SD, standard deviation; AAR, AST to ALT ratio.

Figure 3 shows FIB-4 changes by age and status of HBV and/or HCV, compared to AAR. The FIB-4 in persons with HBV and HCV positivity was higher than among those with negative diagnoses. AAR was not different between the positive and negative results of HCVAb.

**Figure 4:**
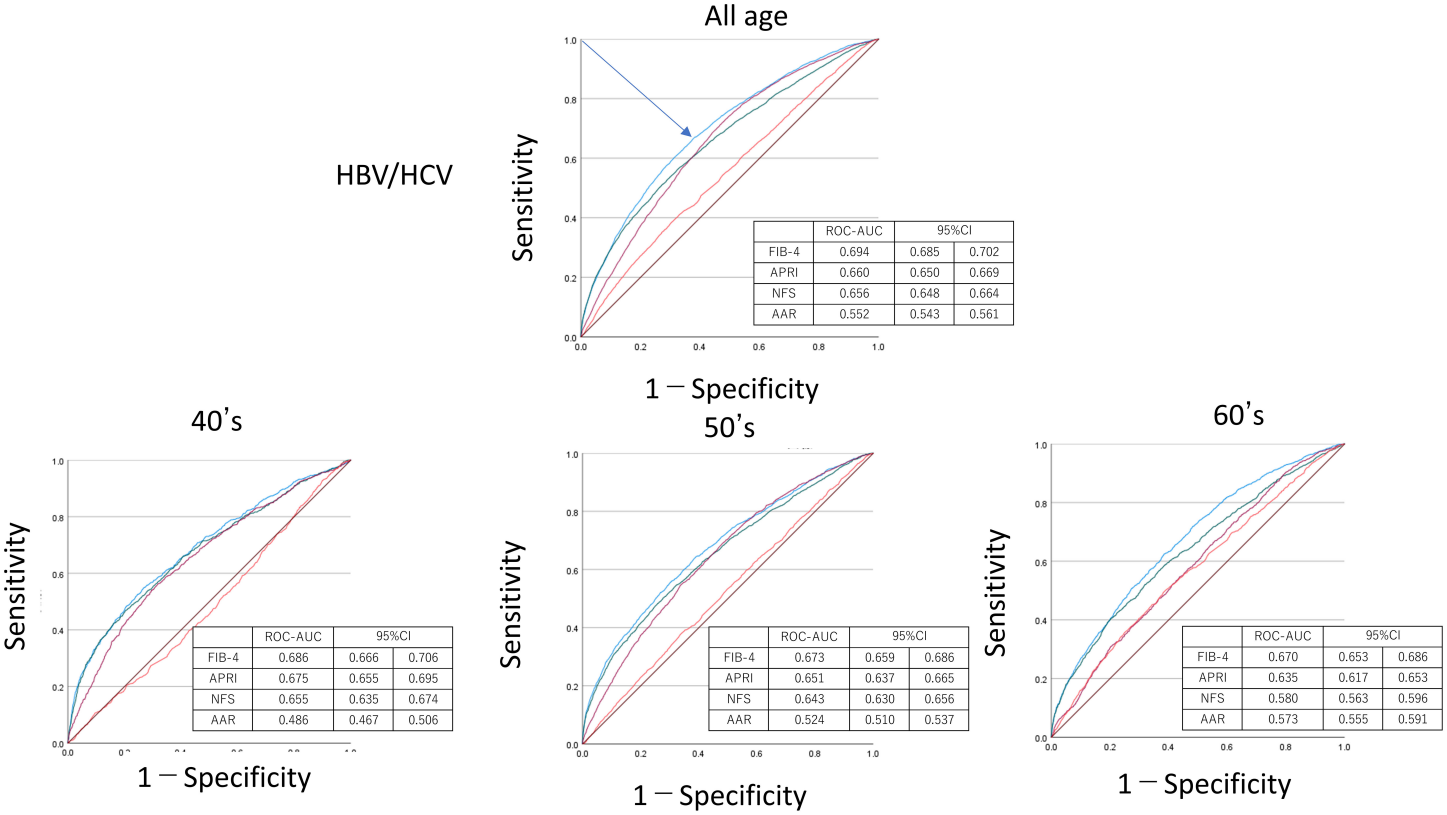
Receiver operating characteristic curve–area under the curve (ROC–AUC) of HBV or HCV by age. ROC–AUC and 95% CI were calculated by FIB-4 (blue), APRI (purple), NFS (green), and AAR (red). Arrow indicates the point on the curve closest to (0.1). CI, confidence interval; APRI, AST to platelet ratio index; NFS, NAFLD fibrosis score; AAR, AST to ALT ratio.

**Figure 5:**
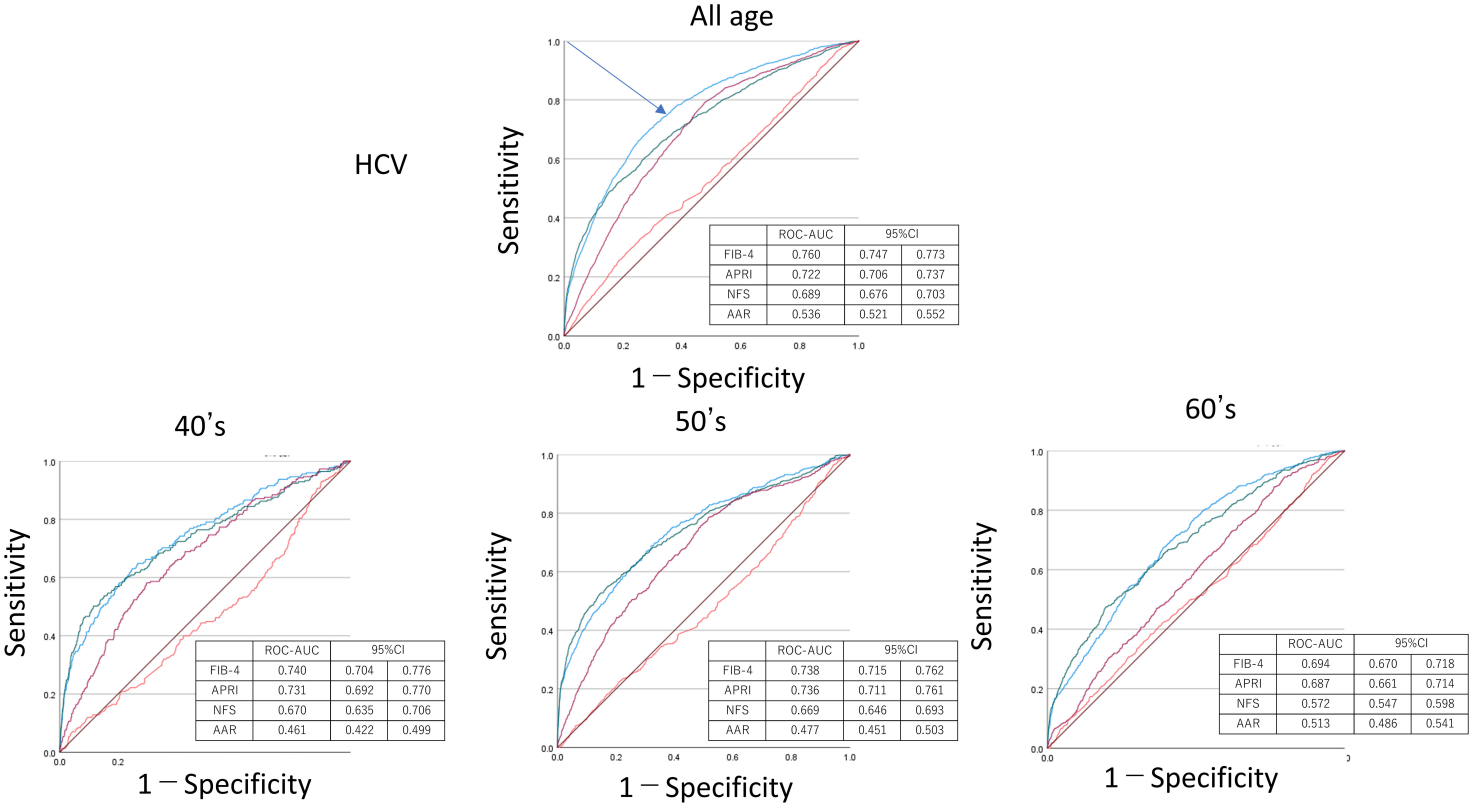
Receiver operating characteristic curve–area under the curve (ROC–AUC) of HCV by age. ROC–AUC and 95% CI were calculated by FIB-4 (blue), APRI (purple), NFS (green), and AAR (red). Arrow indicates the point on the curve closest to (0.1). CI, confidence interval; APRI, AST to platelet ratio index; NFS, NAFLD fibrosis score; AAR, AST to ALT ratio.

**Figure 6:**
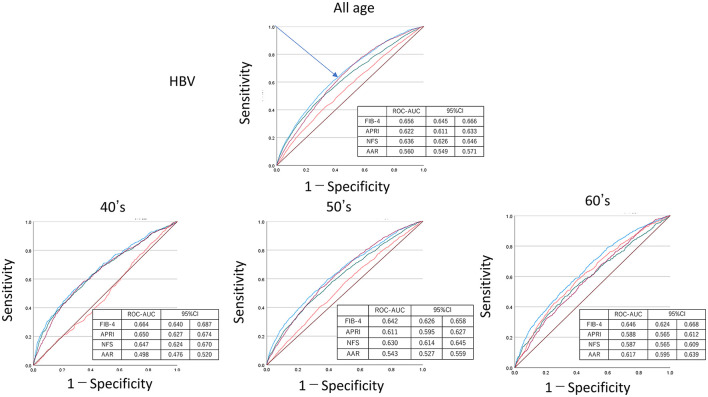
Receiver operating characteristic curve–area under the curve (ROC–AUC) of HBV by age. ROC–AUC and 95% CI were calculated by FIB-4 (blue), APRI (purple), NFS (green), and AAR (red). Arrow indicates the point on the curve closest to (0.1). CI, confidence interval; APRI, AST to platelet ratio index; NFS, NAFLD fibrosis score; AAR, AST to ALT ratio.

Figures 4–6 show the predictive ability of HBV or HCV using ROC analysis by age groups among FIB-4, NFS, APRI, and AAR. The ROC–AUC for detection of HBV/HCV by FIB-4, NFS, APRI, and AAR for all ages was FIB-4 = 0.694, 95% CI = 0.685–0.702; APRI = 0.660, 95% CI = 0.650–0.669; and NFS = 0.656, 95% CI = 0.648–0.664. These indexes, including platelet count, were higher than that of AAR = 0.552, 95% CI = 0.543–0.561. The ROC–AUC by FIB-4, NFS, APRI, and AAR showed a higher detection of HCV than HBV among all ages in FIB-4 = 0.776 (95% CI = 0.747–0.773) vs. 0.656 (95%CI =0.645–0.666); APRI = 0.722 (95% CI = 0.706–0.737) vs. 0.622 (95% CI = 0.611–0.633); NFS = 0.689 (95% CI = 0.676–0.703) vs. 0.636 (95% CI = 0.626–0.646); and AAR = 0.536 (95% CI = 0.521–0.552) vs. 0.560 (95% CI = 0.549–0.571). The sensitivity/specificity of FIB-4 for HVCAb positivity was estimated to be lower than 75/70 at maximum in Figure 5.

**Figure 7:**
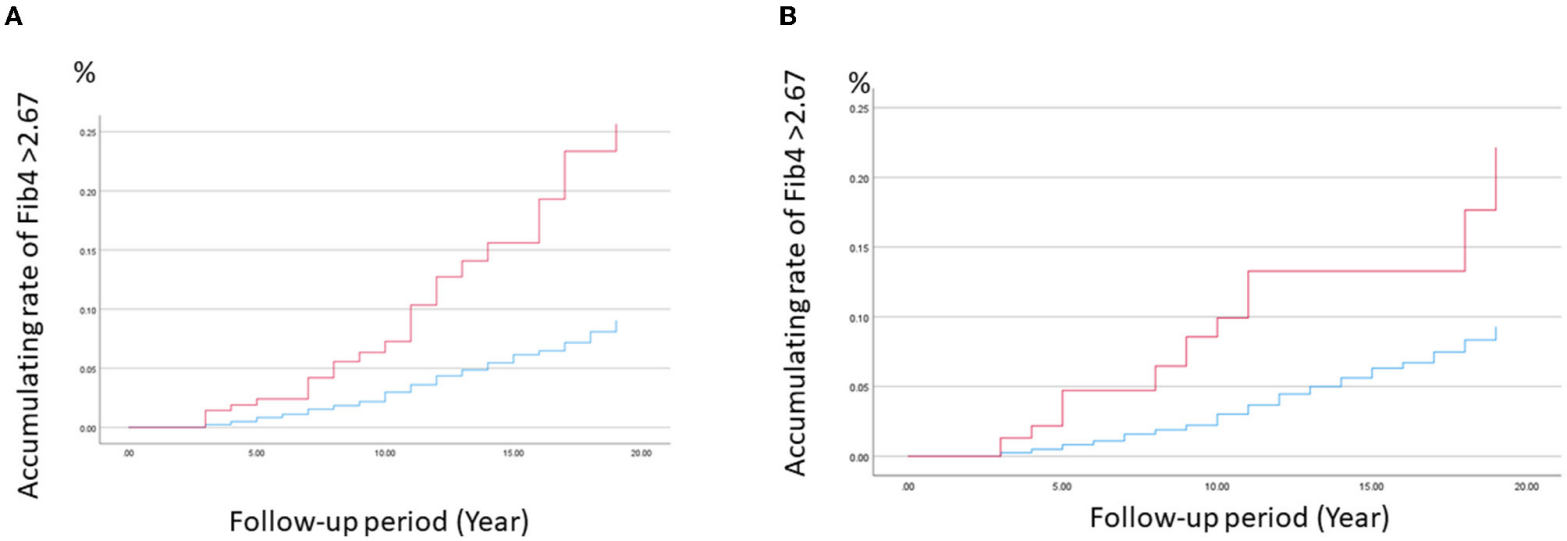
Cumulative ratio of FIB-4 index ≥2.67 for HBV and HCV using the Cox model. The cumulative ratio of FIB-4 index ≥2.67 for **(A)** HBV and **(B)** HCV was calculated using the Cox model among those of positive (red) and negative (blue), after adjusting with age, BMI, and alcohol drinking, respectively.

Finally, related factors were identified as an outcome when the FIB-4 was equal to or exceeded 2.67, according to the Cox method. The results are shown in Figures 7A, B, and Table 3. The HBV and HCV positivity were closely associated with FIB-4 ≥ 2.67 (HBV: HR = 3.03, 95% CI = 2.21–4.15; HCV: HR = 2.95, 95% CI = 2.07–4.20). This was unlikely when liver dysfunction was the outcome, but the fatty liver findings did not show a significant association (HR = 1.11, 95% CI = 0.72–1.71) in model 2.

**Table 3:** The hazard ratio of viral hepatitis test positivity for FIB-4 > 2.67 by Cox proportional models.

	**Crude (age adjusted)**			**Model 1**			**Model 2**		
**Variables**	**HR**	**95% CI**		**HR**	**95% CI**		**HR**	**95% CI**	
HBsAg	3.0	2.0	4.4	3.1	2.0	4.6	3.4	1.9	6.0
HCVAb	2.9	1.9	4.6	3.2	2.0	5.0	2.6	1.0	6.3
Fatty liver^*^							1.1	0.7	1.7

Model 1: HR was adjusted with age, alcohol drinking, and BMI.

Model 2: HR was adjusted with age, alcohol drinking, BMI, and fatty liver.

* Detected by abdominal ultrasonography.

HR, hazard ratio; CI, confidence interval; HBsAg, hepatitis B virus surface antigen; HCVAb, hepatitis C virus antibody.

## Discussion

To the best of our knowledge, such a large-scale study with such long-term data has never been conducted to investigate the significance of FIB-4 on general workers. First, we identified the factors showing a high value of FIB-4 based on the most recent data (dataset-1). As a result, we identified that HCV positivity was mostly associated with FIB-4 ≥ 2.67. Thus, we focused on how FIB-4 could detect viral hepatitis using dataset-2. This dataset included duplicate individuals, and there would be some bias of whether it can be treated as an individual. However, since age was included in the calculation formula for FIB-4, it was treated as an individual case. Finally, we clarified whether viral hepatitis among general workers caused the high level of FIB-4 in individuals using follow-up data for 20 years (dataset-3). These results suggest that FIB-4, including platelet data, might have more predictive potential to detect viral hepatitis than liver function tests alone which are a legal requirement (see text footnote ^1^,^2^).

The measurement of HBsAg and HCVAb, which are straightforward viral hepatitis screening tools, must be widely disseminated and clearly indicated in order to be effective as hepatitis countermeasures. Screening of residents with HBV and HCV infections has been promoted at the national level through the Hepatitis Control Basic Act and a local government project (15). However, hepatitis screening for workers remains insufficient. Many workers undergo annual legal health checkups provided by their employers, but hepatitis virus testing is not a requirement in the workplace in Japan. Since the screening implementation rates for hepatitis B and C have been only 5.2 and 3.8%, respectively (16), and there are still many potential carrier workers (~0.78 million, HBV: 0.48, HCV: 0.3 cases) that remain undiagnosed (16). Using the best FIB-4 cutoff value, the sensitivity/specificity for detecting viral infections was a maximum of 75/75. This sensitivity shows that the importance of HBs antigen and HCV antibody tests for the diagnosis of viral hepatitis cannot be overstated. However, as noted in the Introduction section, the low rate of HBs antigen and HCV antibody tests administered remains a public health issue. The specificity shows that this performance may appear to have the disadvantage of false positives at first glance, hepatitis testing should be conducted at least once in a lifetime, and the findings should be recorded. Even a false positive result is worth confirming a negative result for viral hepatitis, and even a true positive result is of great value in recommending treatment for viral hepatitis. Thus, our result suggests worth considering that usage of information on platelet in legal health checkups might be some help not to overlook workers with hepatitis virus carriers as a complementary countermeasure.

Transaminase (AST, ALT) is an essential measurement item used as a general marker for detecting liver damage in occupational health examinations and legal checkups (see text footnote ^1^,^2^). Our results found only a small number of AST and/or AST abnormalities among subjects with virus hepatitis. It is noteworthy that transaminase levels are often within normal ranges, especially in chronic viral hepatitis, and the ALT levels are usually normal in the immune tolerant and inactivating carriers of chronic hepatitis B (17, 18), chronic hepatitis C, the rate of those having normal ALT levels was reported to be 46–75% (19, 20). Previously, Prati et al. (21) reported that the cutoff of ALT normal range to detect HCV infection was 30 and 19 IU/L, in men and women, respectively. If that value is reduced, liver dysfunction will increase considerably, making post-measurement difficult. Thus, when using the current cutoff value (≥40 IU/L), transaminase abnormalities alone were affected by metabolic syndrome constituent factors and alcohol in health examinations among male workers and were considered to be unsuitable as surrogate markers for viral hepatitis.

The AAR ratio (AST/ALT) was developed as the first indicator of liver fibrosis, reflecting the fact that transaminase has an AST-dominant ascending pattern due to the decrease in normal hepatocytes due to the progression of liver fibrosis (22). Although AAR is effective in excluding diagnoses of more advanced cirrhosis, it is less sensitive in subsequent studies and has not been established as effective in diagnosing lower levels of fibrosis (23). The AAR is a simple marker that does not require a platelet count and can be measured only with legal requirement items. However, our results show that AAR has a low potential to effectively detect asymptomatic workers with viral hepatitis in health examinations.

The FIB-4 and APRI were developed as predictors of fibrosis in hepatitis C (4, 24). Both FIB-4 and APRI showed similar potential to detect hepatitis virus but tended to be higher in FIB-4. The potential to detect hepatitis B tended to be lower than that of hepatitis C. This is because hepatitis B could have various stages among HBsAg-positive cases and contains a high proportion of asymptomatic carriers, thus, there is a difference in the risk of liver fibrosis between HBsAg-positive and HCVAb-positive cases. We investigated whether other markers such as NFS using platelets have a higher hepatitis detection rate. However, it was considered that its power seemed to be lower than that of FIB-4. AAR not including platelet was significantly lower than FIB-4 as mentioned earlier.

In occupational settings, legal health checkups reveal that ~17% of workers have some form of liver dysfunction based on ALT, AST, and/or gamma-glutamyl transpeptidase (γ-GTP) data.^5^ Among workers with liver dysfunction, viral hepatitis should be ruled out first, but in many liver dysfunction cases, viral hepatitis will not be initially diagnosed because patients remain largely asymptomatic until their cases progress (25). Therefore, it is considered that there are still many potential carriers that remain undiagnosed. A recent study found that in 2015 in Japan, over 1.1 and 0.9 million individuals were HBV and HCV carriers, respectively (26). As mentioned earlier, another recent study of ours estimated ~0.78 million (HBV: 0.48, HCV: 0.3) cases to be present among workers (16).

Despite the measurement of HBsAg and HCVAb being simple viral hepatitis screening methods, when Japanese employers conduct viral hepatitis screening tests in workplaces, some problems have arisen, including social discrimination against HBV/HCV-infected people in Japan's working population (27). The risk of HBV and HCV infection from daily contact at work is very low, but previous studies found that 32.1% of people avoided contact with infected colleagues, and 23.7% were prejudiced toward those with HBV and HCV infections because of perceived associations with homosexuality, multiple sexual partners, and drug users (28). In some cases, health examinees do not wish to undergo the examination due to anxiety about the possibility of being disadvantaged by a positive diagnosis. Therefore, hepatitis risk assessment using currently available alternative indicators may facilitate access to hepatitis testing for some of these individuals. This is especially true for the HCVAb test, which is not included in the basic recommendations of the Japan Society of Ningen-Dock,^6^ whose comprehensive medical examination criteria are well-established in Japan.

Since 2014, the treatment of viral hepatitis has made great strides with recent drug development, and antiviral therapy has helped to achieve long-term suppression of HBV replication, allowing most patients with HCV to be treated (29, 30). Despite these advancements, the liver disease still accounts for ~2 million deaths annually worldwide, and hepatocellular carcinoma (HCC) and cirrhosis are global health problems (31). Viral hepatitis remains the main cause of HCC and liver cirrhosis, even in Japan (32, 33).

The strength of this study is that the significance of the FIB-4 index was examined both in cross-sectional and longitudinal studies using a large population. Limitations of this study include that there have been problems with the accuracy of infection diagnoses in analyses that were performed only for men, and the presence of the virus was not confirmed by RNA for the HCVAb. Information on treatment for hepatitis was lacking. Moreover, measurement errors and non-hepatic factors, including thrombocytopenia and hyperthrombocytosis in hematologic diseases, can cause issues with platelet counts. Differential diagnoses are required in these cases. In the longitudinal study, which takes 20 years to conduct, the possibility of measurement biases resulting from the replacement of measuring equipment cannot be denied. The sensitivity/specificity of FIB-4 for HCVAb positivity was up to 75/70 at maximum from the ROC curve as a screening. Considering the prevalence of viral hepatitis, positive predictive values would be low. Thus, it cannot use as a routine screening, and it is necessary to conduct further studies on how to practically use FIB-4 for health management. However, despite these limitations, this study found that high FIB-4 levels could be motivated to refer workers with a high value to hepatologists.

Certainly, it is most efficient to carry out specific virus tests, such as HBsAg and HCVAb for all employees annually until reaching 50 years of age. In addition, we should strive for the social implementation of measures aimed at eradicating hepatitis. Our study indicates that during the implementation of these measures, in cases when FIB-4 shows a high value, hepatitis testing should be considered by a hepatologist. Therefore, we propose that the platelet counts, which are collected but intentionally unused for health management among workers in legal health checkups, should be reconsidered.

## Data availability statement

The datasets presented in this article are not readily available because the datasets used in this study are not publicly available due to restrictions under the license for the current study. Requests to access the datasets should be directed to MT, tatemichi@tokai-u.jp.

## Ethics statement

The studies involving human participants were reviewed and approved by Institutional Review Board for Clinical Research, Tokai University (20R369), and the Hitachi Review Board (2021-16). Written informed consent for participation was not required for this study in accordance with the national legislation and the institutional requirements.

## Author contributions

MK and MT acquired funding and received funding acquisition. YW, THo, TN, and THa collected the data. YF performed data cleaning. KK, KF, YF, MK, and MT designed this study. KK, KF, YF, and MT analyzed the data. KK, KF, and MT wrote the manuscript. MK and THa supervised this study and provided critical comments. All authors have reviewed the manuscript. All authors contributed to the article and approved the submitted version.

## Footnotes

^1^*Ordinance on Industrial Safety and Health* Available online at: https://elaws.e-gov.go.jp/document?lawid=347M50002000032.

^2^*Contents of Special Health Checkups*. Available online at: https://www.e-healthnet.mhlw.go.jp/information/metabolic/m-04-005.html.

^3^*Fiscal 2020 Implementation Status of Specific Health Checkups and Specific Health Guidance*. Available online at: https://www.mhlw.go.jp/stf/newpage_03092.html.

^4^*Nationwide “Nigen Dock” Report*. Available online at: https://www.jstage.jst.go.jp/article/ningendock/30/4/30_750/_pdf.

^5^*Legal Health Check Result Report*. Available online at: https://www.e-stat.go.jp/stat-search/files?page=1&layout=dataset&toukei=00450211&tstat=000001018638&stat_infid=000032116572.

^6^*Basic Test Items of Japan Society of Ningen Dock*. Available online at: https://www.ningen-dock.jp/en/other/inspection.
